# Fostering Multidisciplinary Solutions in Aging: The Research Centers Collaborative Network

**DOI:** 10.1093/geroni/igaa057.2974

**Published:** 2020-12-16

**Authors:** Stephen Kritchevsky, Odette van der Willik, Basil Eldadah

## Abstract

The important problems facing older adults will not be solved through the methods of a single discipline. In recognition of this, the NIA funded the Research Centers Collaborative Network (RCCN) to build collaborations between scientists from the 6 NIA-sponsored center programs: Alzheimer’s Disease Research Centers, Centers on the Demography and Economics of Aging, Claude D. Pepper Older Americans Independence Centers, Nathan Shock Centers of Excellence in the Basic Biology of Aging, Resource Centers for Minority Aging Research, and Roybal Centers for Translational Research on Aging. RCCN’s central premise is that researchers from different disciplines are most likely to collaborate when they are addressing similar problems. To foster collaboration the RCCN has convened 5 workshops on: 1. achieving and sustaining behavior change in older adults; 2. sex and gender in aging research; 3. reserve and resilience; 4. life course perspectives on aging; and 5. promoting the inclusion of older adults in clinical research. After each Workshop the RCCN awards pilot funds related to the theme. This symposium will review key learnings from the workshops and present work of four RCCN pilot teams from the first two workshops which focused on changing and sustaining behavior change in older adults, and sex and gender differences in aging. Dr. Hughes will discuss the value of interdisciplinary research to maintain behavior change while Dr. Lee will discuss social incentives to improve mobility postoperatively. Dr. Stites will discuss cognition and gender trends, while Dr. Ware will discuss sex differences in genetic effects.

